# Now for the hard ones: is there a limit on CRISPR genome editing in crops?

**DOI:** 10.1093/jxb/erz007

**Published:** 2019-02-04

**Authors:** Jose R Botella

**Affiliations:** Plant Genetic Engineering Laboratory, School of Agriculture and Food Sciences, University of Queensland, Brisbane QLD, Australia

**Keywords:** *AP3*, CRISPR/Cas9, flowering, *Fragaria×ananassa*, *Fragaria vesca*, genome editing, MADS-box transcription factors, strawberry, *TM6*

## Abstract

This article comments on the following paper:

**Martín-Pizarro C, Triviño JC, Posé D.** 2019. Functional analysis of the *TM6* MADS-box gene in the octoploid strawberry by CRISPR/Cas9-directed mutagenesis. Journal of Experimental Botany 70, 885–895.

**CRISPR/Cas9-mediated genome editing could revolutionize agriculture but many of the most important (and sometimes most delicious) crops are polyploid adding significant complexity. Martín-Pizarro *et al.* (2019) used CRISPR/Cas9 to edit the genome of the octoploid and highly heterozygous cultivated strawberry (*Fragaria* × *ananassa* cv. Camarosa). They provide functional evidence that *FaTM6*, previously annotated as *FvAP3* in the diploid *Fragaria vesca*, is instead a member of the *TM6* lineage of floral class B homeotic genes and critical to anther and pollen development. Striking phenotypes in the T0 generation prove that CRISPR/Cas9 can be successfully applied even to the most difficult crops.**

The existence of genetic diversity is critically important in crop improvement programmes and new beneficial traits are continuously sought in wild relatives of modern crop species. Nevertheless, identification of the genes associated with those traits is a difficult task and introgression of the trait into elite varieties is time consuming and labour intensive. As an alternative to the use of naturally occurring genetic variation, breeders have used random mutagenesis methods for decades in mutation-based breeding programmes, delivering over 3000 commercial varieties in more than 230 different crops and plant species (Oladosu *et al.*, 2016). Despite its obvious pitfalls, the use of this stochastic approach made sense at a time when genomic information was scarce and sequence data on quantitative trait loci (QTLs) was almost non-existent. Today, with the advent of new-generation sequencing technologies, identification of important QTLs is proceeding at an unprecedented rate (Zhu *et al.*, 2017) and a more precise intervention approach is needed. Gene editing provides the means to target individual loci with high precision although the technically complicated nature of the early technologies, such as zinc finger nucleases, limited its adoption to a few select research laboratories for a long time (Zhang *et al.*, 2017).

## CRISPR/Cas9 is bringing gene editing to every home

Gene editing is based on the ability of certain proteins to recognize a specific DNA sequence and subsequently produce single- or double-strand breaks at that specific position (Zhang *et al.*, 2017). The nuclease activity to produce the breaks is relatively easy to achieve but the recognition of the DNA sequence was the Achilles’ heel of the approach. DNA recognition by amino acid-based protein domains requires the assemblage of large molecules in order to recognize the minimal 19–23 bp sequence needed to provide selectivity. The CRISPR/Cas9 system overcomes the problem by using a two-component system in which DNA recognition is performed by an RNA molecule using base complementarity and the double-strand break is performed by an associated nuclease. The technical simplicity of the CRISPR/Cas9 system makes construct preparation extremely fast with cloning routinely achieved in our laboratory in one afternoon (after designing and synthesizing the necessary oligonucleotides). As a result, genome editing has gone from being the exclusive realm of very specialized research groups to being used in literally thousands of laboratories for routine genome mutagenesis purposes.

## Practical problems in genome editing of elite crop varieties

Shortly after the first reports of genome editing using CRISPR/Cas9 were published in animal systems, the plant community enthusiastically embraced the new technology. The standard method involves the generation of transgenic lines containing the CRISPR/Cas9 expression cassette integrated into the genome. Once mutations are produced and detected, the transgenes can easily be removed by ordinary Mendelian segregation via backcrossing with the parental line (Mao *et al.*, 2017). This approach has been used in a number of crops with outstanding success but its application is far from universal. Many crops such as cotton are recalcitrant or have very difficult and long transformation protocols (Gao *et al.*, 2017) and for some others there is no transformation method available. Even for crops where a transformation method has been established, such as sorghum, many of the elite varieties remain uncooperative, not being amenable to transformation. In the case of the highly heterozygous and self-incompatible pineapple, removal of the transgene cassette is almost impossible. The development of non-transgenic approaches using transient expression of nucleic acids or ribonucleoproteins can solve some of these issues (Zhang *et al.*, 2016*a*; Liang *et al.*, 2017). In addition to the already-mentioned problems, polyploid crops require increased efficiency in order to simultaneously target multiple alleles.

## Solutions with cultivated strawberries (and other disadvantaged species)

Cultivated strawberry (*Fragaria* × *ananassa*) poses a number of problems for precise gene editing. First, the genome of *F.* × *ananassa* is not yet available although fortunately the diploid wild strawberry *F. vesca* has been fully sequenced (Shulaev *et al.*, 2011) and can be used as a reference to facilitate studies in *F.* × *ananassa*. *Fragaria* × *ananassa* cv. Camarosa is not only octoploid but also displays a high level of heterozygosity among alleles. Martín-Pizarro *et al.* (2019) used the *F. vesca* reference genome to gain information about the allelic variation in the *FaTM6* locus. Although not a perfect solution, PCR amplification of the targeted genomic locus followed by sequence analysis of the amplicons can provide a good idea of the sequence differences among alleles. In the case of *F.* × *ananassa* at least five different alleles were identified for the *FaTM6* locus. To circumvent the sequence variations among the different alleles it is wise to use more than one target within the gene by co-expression of two or more sgRNAs. There are a number of groups that have used multiple sgRNAs to simultaneously target up to six different genes/loci (Zhang *et al.*, 2016*b*) and there is no reason why the same strategy cannot be used to target different alleles. Aside from increasing the overall mutagenesis efficiency, this approach has the added benefit of being able to produce fragment deletions between the two targeted sites.

In the case of strawberry, Martín-Pizarro *et al.* (2019) decided to pursue a dual sgRNA approach with one of the target sequences being conserved in all alleles (sgRNA#2) while the other (sgRNA#1) was conserved in four out of the five alleles, containing a single mismatch for the remaining allele (allele #5). The presence of mismatches in the targeting sequence seriously diminishes the cleavage efficiency of the sgRNA/Cas9 ribonucleoprotein although the extent of the reduction depends on the position of the mismatch (Anderson *et al.*, 2015). In cases such as cultivated strawberries, the high level of heterozygosity might require the use of ‘imperfect’ targets in some cases, therefore it is important to determine the effect of the mismatch in the generation of CRISPR/Cas9-induced mutations. In fact, no mutations caused by the sgRNA#1 in allele #5 were detected, reinforcing the importance of maximizing the chances of success by using multiple sgRNA targets.

Genetic transformation of *F.* × *ananassa* is not easy; it can be accomplished in 6–9 months but the efficiency is not as high as other species resulting in the generation of a limited number of transgenic lines. This makes it important to determine in advance the efficiency of the sgRNAs selected for the CRISPR/Cas9 system, as the sequence of the target site has a strong influence on the efficiency of the sgRNA (Wang *et al.*, 2014). Agroinfiltration is widely used in plants for transient expression studies and has been applied for CRISPR/Cas9 target validation in hard-to-transform crops such as cotton (Gao *et al.*, 2017). Fortunately this technique works well in strawberry fruits and can be applied before investing time, effort and money to produce stable transformants. As an alternative, transfection of protoplasts can provide an idea of the sgRNA efficiency (Zhang *et al.*, 2016*b*).

## Even octoploid highly heterozygous species can be mutagenized with CRISPR/Cas9

Molecular analysis of three independent T0 transgenic lines showed the presence of multiple kinds of mutations in the different *FaTM6* alleles, mostly typical small indels. Interestingly, as intended by the authors, large deletions (~190 nt) of the genomic region located between the two target sites were also observed as a result of simultaneous double-strand breaks. The ability to produce CRISPR/Cas9-mediated mutations in the T0 generation of high ploidy crops is truly important as it saves time, effort and money in functional characterization studies. A suggested workflow for the application of CRISPR/Cas9-mediated mutagenesis to difficult crops is provided in Box 1. However, let’s not forget that CRISPR is merely a tool and the ultimate goal is not just to produce a mutation, but to characterize the effects of such mutation, in this case alterations in flower development.

Box 1. **A workflow for CRISPR/Cas9-mediated mutagenesis in ‘disadvantaged’ plant species**

Working with ‘problematic’ species—lacking genome sequence, high ploidy, high heterozygosity, or a combination—requires scientific ingenuity. If the genome is available, the path is relatively straightforward with the first step being the detection of allelic variants by PCR amplification of the intended gene target followed by high-throughput sequencing of the amplicons. It is important to remember that reference genomes will have sequence differences with whichever seed stock is being used for the mutagenesis, even within the same variety. Given the detrimental effect in CRISPR/Cas9 efficiency caused by mismatches in the target sequence, it is vital to confirm the sequence of the targeted regions. Once allelic variants have been identified, bioinformatics analysis can be used to find CRISPR/Cas9 target regions common to several alleles. If possible, it is advisable to target each allele with at least two sgRNAs to improve the chances of success in the T0 generation. Several multiplex CRISPR/Cas9 vectors for plants are available (Zhang *et al.*, 2016*b*). It is also a good strategy to verify the efficiency of the sgRNAs using transient transformation methods before investing months of work in the stable transformation. If the genome is not available for the species of interest, the main obstacle is collecting sequence information about the allelic variants. The existence of genome information in related species can help, as it did in the case of cultivated strawberry, otherwise sequence information will need to be obtained by alternative methods such as next-generation sequencing with limited bioinformatics analysis focused on the locus of interest. Alternatively, imaginative solutions such as the use of conserved protein motifs to design degenerate primers for PCR could also help to amplify the genomic alleles for the gene of interest (Cazzonelli *et al.*, 1998).

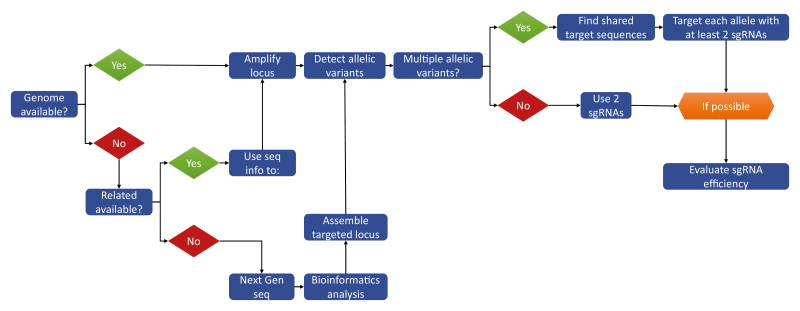

## The good old ABC model of flower development

The ABC model of flower development (Bowman *et al.*, 1991; Coen and Meyerowitz, 1991) is in my view one of the most elegant mechanistic models in developmental biology and living proof that human genius is superior to technological advances (Bowman *et al.*, 2012). Over the 27 years after its initial publication, many refinements have been added but the quintessential message is the same: floral meristem developmental fate is dictated by the action of three classes of genes: A, B and C. Different combinations occur in each of the concentric whorls of a flower with class A being active in the outermost two whorls (1&2), class B in the intermediate ones (2&3) and class C in the innermost (3&4) whorls. As in any family, not everything is in harmony and class A and C members do not get along, negatively regulating each other. In Arabidopsis, class A function is provided by APETALA1 (AP1) and APETALA2 (AP2) and their activity results in the formation of sepals in whorl #1. Class B function is provided by APETALA3 (AP3) and PISTILLATA (PI) and, when working in combination with class A activities, directs the formation of petals in whorl #2. Finally, class C activity is provided by AGAMOUS (AG), which produces stamens in whorl #3 when working in combination with class B activities and carpels when working alone in whorl #4.

Interestingly, the two model systems that were used to formulate the ABC model, Arabidopsis and *Antirrhinum majus*, have a single *AP3* gene while in most of the remaining plant species the ancestral *AP3* gene was duplicated into two distinct phylogenetic lineages, known as *euAP3* and *Tomato MADS box gene6* (*TM6*), with somewhat different functions (Kramer *et al.*, 1998; de Martino *et al.*, 2006; Rijpkema *et al.*, 2006).

## Two class B AP3 homologues, one retaining typical functions of the TM6 lineage

Functional studies in cultivated strawberry are inherently difficult due to the high ploidy of the crop and are further complicated by the high heterozygosity present in the four pairs of homoeologous chromosomes. Martín-Pizarro *et al.* (2019) describe the functional characterization of an AP3 homologue in cultivated strawberry, previously annotated in *F. vesca* as *FveAP3* (FvH4_1g12260 or gene14896-v1.0-hybrid) (Hollender *et al.*, 2014). Phylogenetic analysis showed that *F. vesca* contains one member for each of the two AP3 lineages and, despite its previous annotation, FvH4_1g12260 falls within the TM6 lineage. FvH4_1g12260 expression patterns are consistent with the phylogenetic classification but final confirmation can only be reached after functional characterization of the gene.

Phenotypic characterization of three independent CRISPR/Cas9-generated mutant lines showed relatively small effects on petal size and colour but a very severe effect in anther development with up to 50-fold reduction in pollen number and an almost complete loss of viability in the pollen of mutant lines. All these phenotypes are consistent with previously reported TM6-silenced lines of tomato and *Populus trichocarpa*, thus confirming the identity of FvH4_1g12260 as FvTM6. Although most of the available studies have focused on *euAP3*, it seems that in most species where the ancestral *AP3* gene has been duplicated, there has been a functional differentiation process in both proteins; this means *euAP3* members having a more pronounced role in petal and stamen development, while TM6 homologues appear to be most prominent in anther/pollen development, although they have retained a minor role in petal development.

## Now for the hard ones

On the occasion of the publication of the first plant genome, *Arabidopsis thaliana*, David Adam wrote an insightful commentary—‘Now for the hard ones’—emphasizing the need to persevere and apply the then nascent genome sequencing technology to important crops, regardless of the difficulties posed by those genomes (Adam, 2000). Today, most of the ‘impossible’ genomes are available and the newly acquired knowledge has been invaluable. The same needs to happen with CRISPR/Cas9. After the initial examples in ‘easy’ model species, and in order to make CRISPR/Cas9 a truly universal technology for crop improvement, scientists must keep focusing their attention on complicated crops. Hexaploid bread wheat has now been successfully edited (Zhang *et al.*, 2016*a*; Liang *et al.*, 2017) and Martín-Pizarro *et al.* (2019) go one step further. It seems that genome editing is here to stay and will not be limited by genome complexity.
